# Mediating effect of self-esteem on the relationship between leisure experience and aggression

**DOI:** 10.1038/s41598-022-14125-w

**Published:** 2022-06-14

**Authors:** Ximei Xia, Xiaotian Wang, Hairong Yu

**Affiliations:** Department of Psychology, Qingdao University, Qingdao, China

**Keywords:** Psychology, Human behaviour

## Abstract

Previous research has shown that both the daily experiences and personal traits of adolescents are linked to aggression. Our aim was to further investigate the relationship between leisure experience, self-esteem, and aggression according to the general aggression model. In addition, within frustration-aggression theory, we proposed that leisure experience and aggression have a negative correlation. Furthermore, based on broaden-and-build theory, we explored the mediating role of self-esteem between leisure experience and aggression. The participants included 660 Chinese teenagers with an average age of 14.3. Among them, male students accounted for 310 (49.4%) and female students accounted for 318 (50.6%). The results showed that leisure experience was positively correlated with self-esteem and negatively correlated with aggression, while self-esteem was also negatively correlated with aggression. Additionally, self-esteem fully mediated the relationship between leisure experience and aggression. Our study could enrich research on leisure and provide a basis for protective factors of aggression in adolescents.

## Introduction

Aggression is a type of anti-social behavior that is performed with the purpose of harming other individuals or groups. It basically appears in the forms of physical, verbal or relational aggression^1^. According to an American study, 23.6% of adolescents in Grades 9–12 reported experiencing at least one incident of physical conflict within a year^2^. Globally, interpersonal violence is the fourth leading cause of death among 15–19-year-olds, with a mortality rate of 5.5%^3^. On the one hand, levels of aggression may increase during adolescence^4^. On the other hand, both perpetrators and victims of aggressive behaviors may suffer long-term negative consequences. In previous studies, engagement in aggressive behaviors is associated with an increased risk of future negative socioeconomic consequences and participation in later violent and nonviolent crimes^5,6^. In addition, victims are likely to experience mental health problems^7^. Therefore, in this study, we expected to explore factors that might be related to aggression in adolescents.

The general aggression model states that personal and situational factors influence the ultimate aggressive behaviors through the present internal state^8^. In general, aggression can be displayed and learned in a variety of settings, including family, school, and daily recreation^9–11^. As a situational factor, satisfying leisure experience was found to be negatively associated with aggression^12^. As an individual trait, lower self-esteem is also closely related to aggression^13,14^. However, as far as we are concerned, no study has investigated the relationship between leisure experience, self-esteem and aggression, which is the main goal of this study.

### Leisure experience and aggression

Leisure experience refers to the participants’ perceived significance of the leisure activity, which is a construct with a theoretical tradition and practical application^15^. The perception of freedom, competence, intrinsic motivation, relatedness and control are central to many leisure experiences^16–18^. We primarily focus on perceived freedom, perceived competence, and perceived intrinsic motivation in our research, considering that these three kinds of experience are closely related^19^.

As a period of nonobligatory time, leisure, in which people seek marvelous experiences of self-determination, is thought of as compensation for work^16^. In a sense, leisure time without the perception of freedom, competence and intrinsic motivation is probably considered a failure^20,21^, which has a link to the feeling of depression, anxiety and distress^22,23^. Within the framework of frustration-aggression theory, frustration, defined as irritable distress from limitation, exclusion and failure, may provoke defensive or aggressive behavioral responses^24^. From this perspective, positive leisure experience is likely to be negatively associated with aggression. As research has demonstrated, wonderful experiences in leisure time have a correlation with reduced aggression^25^. Accordingly, we propose the following hypothesis:

#### **Hypothesis 1**

Adolescents' leisure experience is negatively correlated with aggression.

### The mediating role of self-esteem

Self-esteem is an individual's self-assessment that reflects how much he appreciates himself^26^. Traditionally, lower self-esteem may be mutually related to certain negative outcomes, such as externalizing problems such as aggression^27^. As some scholars have argued, individuals with lower self-esteem may act aggressively to avoid feelings of inferiority brought on by failure^28,29^. Additionally, a meta-analytical study of Chinese students found a moderate negative correlation between self-esteem and aggression^30^. Based on this evidence, we consider that self-esteem as an individual cognitive factor might be negatively correlated with aggression.

Moreover, broaden-and-build theory proposes that experiences of positive emotion may contribute to the construction of higher self-evaluations^31^. As has been proven, positive feelings can build lasting personal resources, including self-acceptance and self-esteem^32^. Similarly, a pleasant leisure experience was generally positively associated with self-esteem in the former research. For instance, leisure satisfaction has been shown to have a positive impact on self-esteem^33^. Research has also confirmed that engaging in gratifying entertainment programs is positively associated with self-esteem and self-concept^34^.

Therefore, in the aforementioned case that both leisure experience and self-esteem may relate to aggression, we additionally propose that the relationship between leisure experience and aggression could be mediated by self-esteem. Self-esteem has often been used as a mediator between different experiences and behavioral problems among adolescents, for example, ostracism and aggression^35^, school disconnectedness and Internet addiction^36^, and peer victimization and problem behaviors^37^. In summary, according to the related findings of leisure experience, aggression and self-esteem, we propose the second hypothesis:

#### **Hypothesis 2**

Self-esteem plays a mediating role between leisure experience and aggression.

## Materials and methods

### Sample and procedure

Participants included 660 students recruited from two high schools and two junior high schools in a mid-sized city of China. Participants were volunteers who were told the content of the questionnaires and agreed that the data could be used in the study. We collected 628 students (average age = 14.3) after removing invalid questionnaires (blank, missing more than five questions or having more than five repeat options). Among them, there were 310 (49.4%) male students and 318 (50.6%) female students. In addition, there were 300 (47.8%) junior high school students and 328 (52.2%) high school students.

This research was conducted from March 2021 to June 2021. Our research team members and one teacher from each school of participants were trained in advance to ensure the quality of the questionnaires. The experimenters first read the instructions and the principle of confidentiality and subsequently organized students to answer the questionnaires. The answering time was limited to 20 min.

### Measures

#### Leisure experience

**Figure 1:**
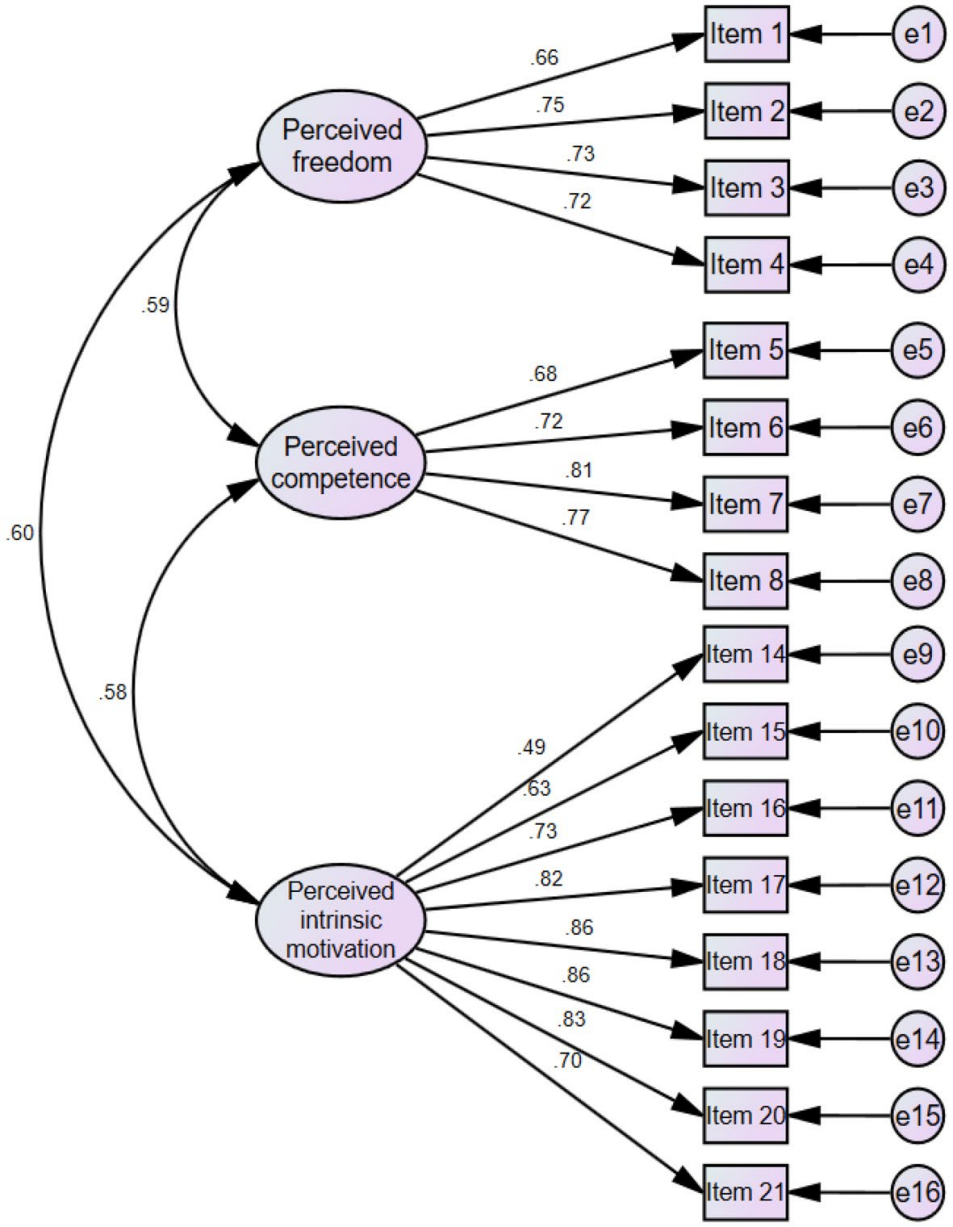
Factor loadings and factor intercorrelations for leisure experience.

This study used the leisure experience scale developed by Hairong Yu^38^. The scale consists of four factors: perceived freedom, perceived intrinsic motivation, perceived competence, and perceived extrinsic motivation. We used the first three factors to assess the leisure experience of adolescents. Among them, perceived freedom includes four questions, such as "I can freely spend my leisure time." Perceived intrinsic motivation includes eight questions, such as "I find a lot of fun in leisure activities." In addition, perceived competence contains four questions, such as "I think leisure activities can improve some of my abilities." In this study, the Cronbach's alpha of the total scale was 0.91, and the Cronbach's alphas of the three subscales were 0.81 for perceived freedom, 0.90 for perceived intrinsic motivation, and 0.83 for perceived competence. In addition, χ^2^/df = 4.52, CFI = 0.93, TLI = 0.92, GFI = 0.91, NFI = 0.92, AGFI = 0.88, and RMSEA = 0.075. We specified the SEM of this scale in Fig. 1, in which the factor loadings and factor intercorrelations are provided. Using a four-point Likert scale, the participants were asked to choose from 1 (completely inconsistent) to 5 (completely consistent). A higher score indicates a higher level of leisure experience.

#### Aggression

This study adopted the Chinese version of the Buss–Perry aggression questionnaire^39^. The scale consists of 20 items, which are categorized into four dimensions: physical aggression, anger, hostility, and substitution aggression. The scale was rated on a four-point Likert scale. The participants were asked to choose from 1 (totally disagree) to 5 (totally agree), with a higher score indicating a higher tendency of aggression. In this study, the Cronbach's alpha of the total scale was 0.88, and the Cronbach's alphas of the four dimensions were 0.77 for physical aggression, 0.85 for anger, 0.81 for hostility, and 0.79 for substitution aggression.

#### Self-esteem

We used the self-esteem scale (SES) in this study^40^. This scale comprises 10 items, five of which are reverse scored. Using a four-point Likert scale, the participants were asked to choose from 1 (completely inconsistent) to 4 (completely consistent). A higher score indicates higher self-esteem. In this study, the Cronbach's alpha of the scale was 0.88.

### Statistical procedure

We used SPSS 21.0 and AMOS 24.0 to analyze the data. SPSS was used for descriptive analysis, Pearson correlation analysis and linear regression analysis. To determine the mediating effect of self-esteem, we conducted SEMs using 5000 bootstrap samples and the 95% bias-corrected confidence interval (95% CI) in AMOS. The level of statistical significance was set as *p* < 0.05.

### Ethical statement

Our research was based on the ethical standards in the WMA Declaration in Helsinki and was approved by the research ethics committee of Qingdao University, China. We informed all participants of the study before the test. The research was conducted after the consent of the participants. In addition, our data were anonymized to ensure the privacy of all participants.

## Results

### The common method bias examination

Since data for this study were collected by self-report questionnaires, we used confirmatory factor analysis to test all items for common method bias^41^, and the results show that the model fit did not fit well, χ^2^/df = 10.78 > 3, RMSEA = 0.121 > 0.08, NFI = 0.44 < 0.9, CFI = 0.47 < 0.9. Therefore, there was no serious common method deviation.

### Descriptive statistics and correlations

The means *(M)* and standard deviations *(SD)* for all variables are displayed in Table 1. The results of correlations can be seen in Table 1 as well. As the table shows, there was a positive correlation between leisure experience and self-esteem (*r* = 0.49, *p* < 0.01) and a negative correlation between leisure experience and aggression (*r* = − 0.35, *p* < 0.01). Self-esteem was negatively correlated with aggression (*r* = − 0.71, *p* < 0.01).

**Table 1:** Descriptive statistics and correlation coefficients (*N* = 628).

Variables	*M*	*SD*	1	2	3	4	5	6	7	8	9	10	11
1 Gender	0.51	0.50	1										
2 Perceived freedom	3.98	0.83	− 0.01	1									
3 Perceived intrinsic motivation	3.93	0.86	− 0.03	0.53**	1								
4 Perceived competence	4.41	0.63	− 0.09*	0.50**	0.50**	1							
5 Leisure experience	4.18	0.61	− 0.05	0.79**	0.87**	0.78**	1						
6 Self-esteem	3.56	0.53	− 0.09*	0.42**	0.39**	0.41**	0.49**	1					
7 Physical aggression	1.12	0.24	− 0.09*	− 0.24**	− 0.29**	− 0.19**	− 0.30**	− 0.41**	1				
8 Anger	2.32	0.97	0.12**	− 0.17**	− 0.14**	− 0.21**	− 0.20**	− 0.53**	0.26**	1			
9 Hostility	2.28	0.86	0.03	− 0.22**	− 0.20**	− 0.20**	− 0.25**	− 0.54**	0.27**	0.50**	1		
10 Substitution aggression	1.98	0.86	0.05	− 0.27**	− 0.31**	− 0.34**	− 0.37**	− 0.63**	0.34**	0.46**	0.52**	1	
11 Aggression	1.87	0.56	0.07	− 0.28**	− 0.29**	− 0.31**	− 0.35**	− 0.71**	0.47**	0.82**	0.78**	0.81**	1

*p < 0.05, **p < 0.01. The gender variable was treated as a dummy variable, 0 = male, 1 = female. Leisure experience is the sum of perceived freedom, perceived intrinsic motivation, and perceived competence. Aggression is the sum of physical aggression, anger, hostility and substitution aggression.

### Linear regression analysis

To further explore the relationship between leisure experience and aggression, we performed linear regression tests. We used gender as a control variable, the four dimensions of leisure experience as independent variables, and aggression as a dependent variable. Linear regression analysis was performed using the "enter" method. The results can be seen in Table 2. After controlling for all other leisure experiences, perceived freedom (*β* = − 0.12, *p* = 0.009), perceived intrinsic motivation (*β* = − 0.13, *p* = 0.007), and perceived competence (*β* = − 0.18, *p* < 0.001) were all negatively correlated with aggression. They totally predicted 12% of the variance of aggression.

**Table 2:** Regression analysis for effects of leisure experience on aggression (*N* = 628).

	Variables	*β*	*F*	*R*^2^	*ΔR*^2^
Step 1			3.21	0.01	
	Gender	0.07			
Step 2			23.52***	0.13	0.12
	Gender	0.05			
	Perceived freedom	− 0.12**			
	Perceived intrinsic motivation	− 0.13**			
	Perceived competence	− 0.18***			

**p < 0.01, ***p < 0.001.

### The mediating effect of self-esteem on leisure experience and aggression

**Figure 2:**
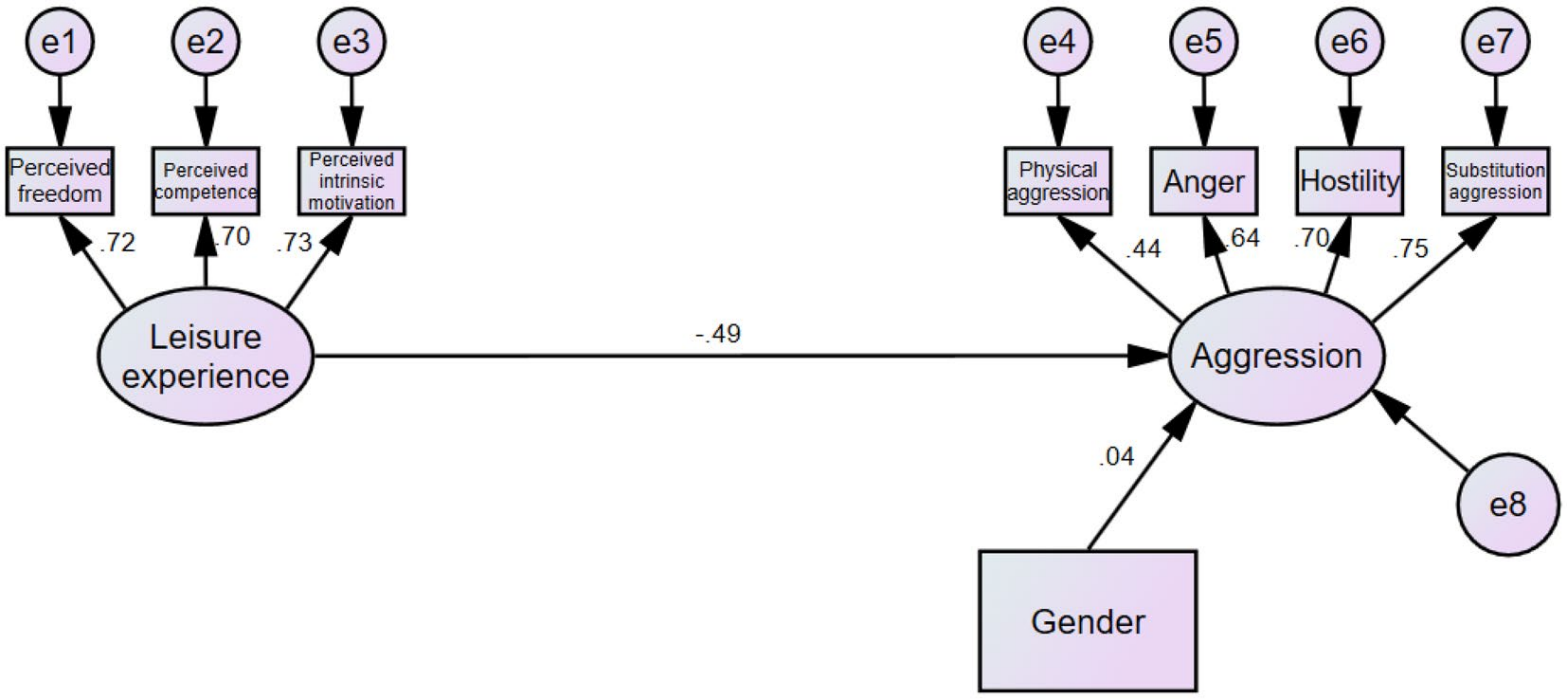
Direct effect of leisure experience on aggression (Model 1).

**Figure 3:**
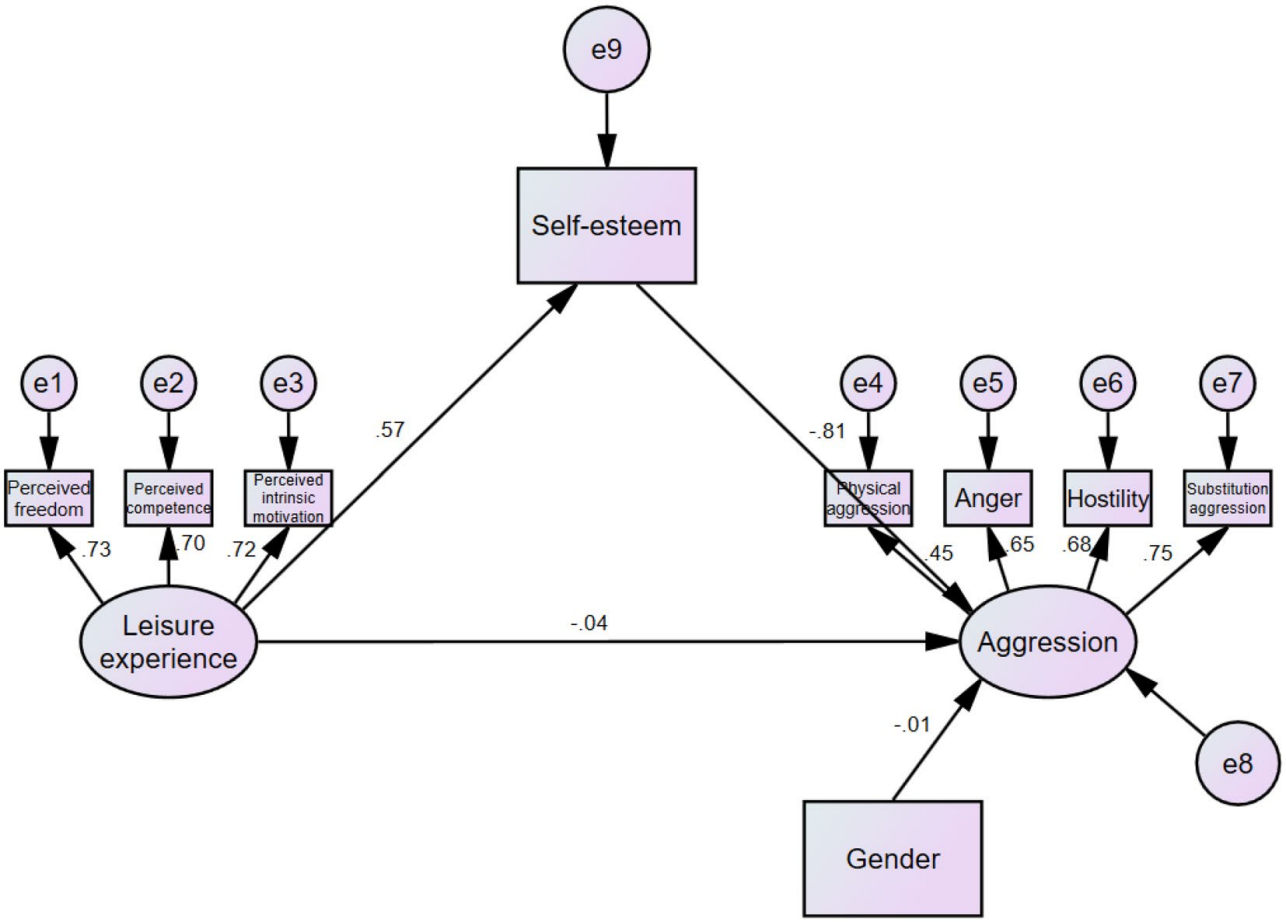
Indirect effect of leisure experience on aggression, mediated by self-esteem (Model 2).

To test the relationship between variables, we used SEMs to estimate two models: (1) a model that includes only direct paths and (2) a model that contains both direct and indirect paths. We considered leisure experience as the independent variable, self-esteem as the mediating variable, aggression as the dependent variable, and gender as the control variable to conduct two models in AMOS. The results are shown in Figs. 2 and 3.

In model 1, leisure experience was significantly negatively associated with aggression (*β* = − 0.49, *p* < 0.001). However, in model 2, after self-esteem (mediating variable) was added, the correlation between leisure experience and aggression was no longer significant (*β* = − 0.04, *p* = 0.455). Meanwhile, there was a significant correlation between leisure experience and self-esteem (*β* = 0.57, *p* < 0.001) as well as between self-esteem and aggression (*β* = − 0.81, *p* < 0.001).

Table 3 demonstrates the model fit indicators and the results of the χ^2^ difference test of specific structural models. In Model 1, χ^2^/df = 3.85, CFI = 0.949, RMSEA = 0.069, RMR = 0.028. For Model 2, χ^2^/df = 3.45, CFI = 0.963, RMSEA = 0.069, RMR = 0.022. Furthermore, the χ^2^ difference test shows that ∆χ^2^ (6) = 13, *p* = 0.043, and model 2 was superior to model 1. In model 2, the direct effect value was − 0.031 (95% CI [− 0.128, 0.063], *p* = 0.586), the indirect effect value was − 0.410 (95% CI [− 0.509, − 0.335], *p* = 0.005), and the total effect value was − 0.441 (95% CI [− 0.544, − 0.344], p = 0.009). These results suggest that the relationship between leisure experience and aggression was fully mediated by self-esteem.

**Table 3:** Fit statistics of tested models and result of χ^2^ difference test.

Model type	χ^2^/df	CFI	RMSEA	RMR	∆χ^2^/(df)	∆CFI
1	3.85	0.949	0.069	0.028	13(6)*	0.014
2	3.45	0.963	0.069	0.022		

*p < 0.05.

## Discussion

Our study was intended to examine the relationship between leisure experience, self-esteem and aggression based on the general aggression model. First, on the basis of frustration-aggression theory, we proposed that leisure experience is negatively correlated with aggression, and the results supported this hypothesis. Second, within the broaden-and-build theory, we suggested that self-esteem may play a mediating role between leisure experience and aggression. The results also confirmed this hypothesis. Overall, our study extends the general aggression model and provides certain evidence for the relationship between leisure experience, self-esteem, and aggression.

### Leisure experience and aggression

Consistent with previous research^42^, we found that leisure experience was negatively correlated with aggression. To our knowledge, this is the first time that we have incorporated multiple perceptions of freedom, intrinsic motivation and competence into leisure experience and examined their relationship with aggression. Our research is dedicated to discovering the benefits of leisure for the healthy development of adolescents. On the whole, great leisure experience is often associated with many positive outcomes, such as psychological well-being^43^ and lower levels of stress^44^, and both have a negative connection with externalizing behavioral problems^45^.

On the basis of the linear regression analysis, perceived freedom, perceived intrinsic motivation and perceived competence of leisure experience were all negatively correlated with aggression after controlling for gender. Perceptions of these three kinds of experience are generally associated with adaptive psychosocial functioning^46,47^. Leisure is sometimes seen as compensation for other areas of life, probably because individuals can gain a deep sense of satisfaction in their perceptions of freedom, competence, and intrinsic motivation^48^. Moreover, positive leisure experiences could effectively reduce people's stress and depression^49^, which are often seen as risk factors for aggression^50^.

In contrast, the absence of any of the above experiences may cause frustration, which, according to frustration-aggression theory, could trigger aggression. In a previous study, the classroom environment in which students' sense of autonomy was frustrated encouraged bullying and interpersonal aggression^51^. Another study found that frustration with competence in electronic games indicates a greater likelihood of aggressive behaviors^52^.

An interesting finding was that the direct effect between leisure experience and aggression was no longer significant after the mediator variable (self-esteem) was added. This may be because the leisure experience investigated was recalled by individuals, while self-esteem is a relatively stable personal trait. As a cognitive trait, self-esteem may be one of the key mechanisms explaining levels of aggression.

### The mediating role of self-esteem

Similar to previous studies, we specifically focused on the potential mediating variable between leisure and aggression^53^. Our study found that self-esteem fully mediated the relationship between leisure experience and aggression, suggesting that perhaps certain cognitive factors, such as self-esteem, may play an intermediary role between individuals’ daily experiences and aggression. Self-esteem is widely influenced by the social environment and individuals’ own experiences^54^, and it is also thought to be a possible antecedent of behaviors^27,55^. Some theories suggest that to avoid the lowering of self-concept, individuals may show externally oriented anger after the threat to the ego^56^.

Individuals could derive many psychological benefits from leisure, such as positive self-concept^57^, profiting from the enjoyable experience during free time. During adolescence, a high sense of mastery and autonomy is connected with higher self-esteem^58,59^, and these perceptions are often regarded as essential components of the leisure experience. Furthermore, Ryan and Deci^60^ pointed out that intrinsic motivation is also associated with many positive effects, such as high levels of creativity, energy, and self-esteem. These results support the broaden-and-build theory, in which positive emotions, as accumulated and compounded, gradually become a lasting individual resource. Conversely, negative experience is usually associated with higher levels of aggression and lower self-esteem^61^.

On another aspect, based on our research, self-esteem and aggression are negatively correlated. Numerous studies have demonstrated this result. For instance, Morsünbül^13^ found that self-esteem could predict the levels of aggression during adolescence and adulthood. Self-esteem is an important construct in the process of individual growth, and it frequently plays a protective role in various psychological symptoms^62^. For adolescents, improving self-esteem may help prevent aggressive behaviors^14^.

## Conclusions and limitations

In short, the results supported two hypotheses, which are as follows: (1) leisure experience was negatively associated with aggression; (2) Self-esteem mediated the relationship between leisure experience and aggression. These results extend the general aggression model and provide some evidence for factors might be associated with aggression in adolescents. In our view, the broad benefits that leisure may bring to adolescents are worthy of attention. At the same time, the role of adolescents' intrapersonal traits also needs to be considered.

There are some limitations to our research as well. First, the study was based on cross-sectional data. Nevertheless, the results may differ from those based on longitudinal data. To further verify the relationship between these three variables, we may need long-term follow-up. In addition, adolescents' self-reported aggression may be concealed so that we could use other ways to make the data more accurate, such as adding observations from teachers or parents.

## Data Availability

The data that support the findings of this study are openly available in “OSF” at https://osf.io/fdq4h/. Identifier: 10.17605/OSF.IO/FDQ4H.
